# Pretreatment ^18^F-FDG PET/CT Imaging Predicts the KRAS/NRAS/BRAF Gene Mutational Status in Colorectal Cancer

**DOI:** 10.1155/2021/6687291

**Published:** 2021-06-18

**Authors:** Peng He, Yuan Zou, Jia Qiu, Tianhong Yang, Lei Peng, Xiangsong Zhang

**Affiliations:** ^1^Department of Nuclear Medicine & Guangdong Engineering Research Center for Translational Application of Medical Radiopharmaceuticals, The First Affiliated Hospital of Sun Yat-sen University, Guangzhou, China; ^2^Department of Ultrasound Medicine & Ultrasonic Medical Engineering Key Laboratory of Nanchong City, Affiliated Hospital of North Sichuan Medical College, Nanchong, China

## Abstract

**Objective:**

To investigate the association between KRAS/NRAS/BRAF mutations and metabolic parameters of pretreatment ^18^F-FDG PET/CT in colorectal cancer (CRC).

**Methods:**

A total of 85 patients with CRC were included in the study. PET/CT was performed in all the patients before surgery. The histopathological examination and analysis of the gene mutational status of the primary tumor were conducted. The associations among clinical features, PET metabolic parameters, and the gene mutational status were investigated. Moreover, receiver operating characteristic (ROC) curves for maximum standard uptake value (SUVmax) of the primary tumor were generated along with analysis of the target tissue to nontarget tissue ratio (T/NT) for predicting the efficacy of KRAS/NRAS/BRAF mutations in CRC. Finally, the corresponding area under the curve, the optimal cutoff value, and the corresponding sensitivity and specificity were obtained.

**Results:**

The mutation rate of KRAS/NRAS/BRAF was 54.12% (46/85). In addition, both SUVmax and T/NT were significantly higher in the KRAS/NRAS/BRAF-mutation groups compared to the wild-type group (15.88 ± 6.71 vs. 12.59 ± 5.79, 8.04 ± 3.03 vs. 6.38 ± 2.80; *P*=0.012 and 0.004, respectively). Results from the ROC curve also showed that the cutoff values for T/NT and SUVmax were 5.14 and 12.40, respectively, while the predictive accuracy was 0.682 and 0.647, respectively. On the other hand, the sensitivity was 91.30% and 65.22% while the specificity was 43.59% and 64.10%, respectively. Moreover, univariate analysis showed that the KRAS/NRAS/BRAF mutation was not significantly associated with gender, age, lesion location, tumor length, pathological type, tissue differentiation, and UICC staging (all *P* > 0.05).

**Conclusion:**

T/NT ratio and SUVmax could be the potential surrogate imaging indicators to predict the KRAS/NRAS/BRAF mutational status in CRC patients.

## 1. Introduction

Colorectal cancer (CRC) is one of the most common malignant tumors, with the third-highest incidence and the second-highest mortality in developed countries [1]. The overall mortality of CRC has declined by nearly 50% over the recent years, as a result of early detection and improved management [2]. The introduction of drugs based on monoclonal antibodies (such as cetuximab and panitumumab) has significantly improved the outcome of patients with CRC [3]. However, some studies revealed that these drugs have low efficacy in patients with KRAS/NRAS/BRAF gene mutations [4–6]. Additional studies also suggested that these mutations may be responsible for the lack of response to monoclonal antibodies targeting the epidermal growth factor receptor (EGFR) [7–10]. Therefore, identification of the KRAS/NRAS/BRAF mutational status is crucial for tailoring personalized treatment strategies and predicting therapeutic options for patients with CRC.

Positron emission tomography and computed tomography (PET/CT) using fluorine-18-fluorodeoxyglucose (^18^F-FDG) are widely used for diagnosis, staging, and postoperative monitoring as well as investigation of metastasis in a variety of cancers [11]. However, evaluating the correlation between pretreatment PET/CT images and genetic changes is a challenging task as it is difficult to optimize the predictive value of the gene mutational status. Presently, pathology is still the gold standard for tumor diagnosis and classification although not all patients can provide a pathological specimen [12]. Therefore, it is important to develop a noninvasive, reproducible method that can reflect intratumoral heterogeneity, to help provide information on the mutational status. Notably, PET/CT may be an ideal tool for this purpose, among the existing molecular imaging techniques [13].

A number of studies [14–18] suggest that glucose accumulation in PET/CT is significantly associated with genetic mutations (KRAS, KRAS/BRAF, or KRAS/NRAS) in primary CRC, metastatic CRC, or rectal cancer. Therefore, PET/CT might be a resourceful, noninvasive imaging method for predicting the gene mutational status of CRC although not all studies found this to be true [19, 20]. Consequently, it is plausible to explore the relationship between different PET/CT metabolic parameters and the gene mutational status in CRC.

In this study, the clinical characteristics and PET/CT imaging parameters of a group of CRC patients were analyzed retrospectively. The potential value of different metabolic parameters in predicting the KRAS/NRAS/BRAF mutational status in CRC was explored. Moreover, the predictive value was verified through the receiver operating characteristic (ROC) curve analysis.

## 2. Materials and Methods

### 2.1. Study Population

The present study retrospectively analyzed 85 patients who underwent PET/CT examination before CRC surgery in the First Affiliated Hospital of Sun Yat-sen University, from January 2017 to May 2020. This study was approved by the Clinical Research Ethics Committee of the hospital (Ethical review approval number: 2019-170). For this retrospective study, the requirement of informed consent was waived. The inclusion criteria were as follows: (i) patients who had not received any prior treatment, including chemotherapy or radiation therapy before the ^18^F-FDG PET/CT scan; (ii) patients with complete clinical information and imaging data; and (iii) patients with CRC confirmed through pathology after surgery and those who had undergone KRAS mutation analysis within 1 month after the PET/CT scan. On the other hand, the exclusion criteria included (i) patients who had received antitumor treatment (e.g., surgery, radiotherapy, and chemotherapy) before PET examination; (ii) patients with incomplete imaging and clinical data; (iii) patients with more than one type of cancers; and (iv) those in which the diagnosis of CRC was not confirmed through pathology. Consequently, 85 CRC patients were enrolled in the analysis, including 51 males and 34 females, with an average age of 59 years (range: 26–79 years). All the patients had a normal serum glucose level before obtaining the PET/CT images. The characteristics of the 85 patients are shown in Table 1.

### 2.2. Acquisition of PET/CT Images

This step used the Gemini GXL 16 scanner (Philips, Netherlands) which integrates a PET scanner with a 16-detector spiral CT that can collect jointly registered CT and PET images in a single inspection. In addition, the ^18^F-FDG tracer was produced by our department using the Cyclone 10 isotope synthesis system from the Belgium IBA Company. The radiochemical purity of ^18^F-FDG was more than 95%. Notably, all the patients were required to fast for at least 6 h and urinated just before starting the PET/CT scan. After entering the waiting room, the patients received an intravenous injection of ^18^F-FDG at a dose of 5.18 MBq (0.14 mCi)/kg and after about 60 minutes, the PET/CT scan was performed from the upper thigh to the base of the skull. The parameters of the noncontrast CT scan were as follows: 120 kV, tube current-time product: 50 to 80 mAs depending on the patient's weight, a slice thickness of 5 mm, and a rotation time of 0.8 seconds. In the same range, PET images were collected in the 3-dimensional acquisition mode and 6-7 beds were scanned in the 90 seconds per bed position. Finally, images were reconstructed with 4 × 4 × 4 mm^3^ voxels using the LOR-Ramla algorithm with low-dose CT images for attenuation correction.

### 2.3. Analysis of PET Images

The PET images were independently interpreted by two senior nuclear physicians, who were unaware of the mutational status. In the semiquantitative analysis, the maximum standardized uptake value (SUVmax) of tumor lesions on the 3-dimensional PET images was measured and 40% of the SUVmax was used as the threshold. Additionally, the computer automatically calculated the SUVmax by manually sketching the region of interest (ROI) in CRC lesions. In cases with multiple lesions, those with the SUVmax were selected as representatives. Moreover, the target tissue to nontarget tissue ratio (T/NT) was expressed as the ^18^F-FDG uptake ratio of the target lesion to the nontarget lesion, and the SUVmax of the primary tumor was selected as the representative value of the target lesion. On the other hand, the glucose uptake value of normal liver parenchyma was selected to represent the nontarget lesion. Therefore, T/NT = tumor SUVmax/liver SUV, where liver SUV was the average value of SUVmax, at three points in the normal liver parenchyma.

### 2.4. Analysis of Genetic Mutations

Pathological samples were obtained following tumor resection and experienced pathologists selected appropriate tumor tissues for analysis. In addition, paraffin-embedded sections were used to extract DNA and the real-time fluorescent quantitative polymerase chain reaction (RT-PCR) technology was employed for amplification detection. Notably, 17 mutations in the KRAS gene (exons 2, 3, and 4), 13 mutations in the NRAS gene (exons 2, 3, and 4), and 1 mutation in the BRAF gene (exon 15: V600 E) were analyzed through a next-generation sequencing (NGS) method. The mutation information of exon 2 of KRAS mutations includes Gly12Ser, Gly12Asp, Gly12Ala, Gly12Cys, Gly12Arg, Gly12Val, Gly13Asp, and Gly13Asp; exon 3 includes Ala59Thr, Gln61Lys, Gln61Leu, Gln61Arg, and Gln61His; and exon 4 includes Lys117Asn, Ala146Thr, Ala146Val, and Ala146Pro. The mutation information of exon 2 of NRAS mutations includes Gly12Ser, Gly12Asp, Gly13Asp, Gly13Arg, Gly12Cys, Gly12Val, Gly12Ala, and Gly13Val; exon 3 includes Gln61Arg, Gln61Lys, Gln61Leu, and Gln61His; and exon 4 includes Ala146Thr.

### 2.5. Statistical Analyses

Data of SUVmax and the T/NT ratio were tested for normality and homogeneity of variance. Thereafter, normally distributed data were expressed as mean ± standard deviation. In addition, quantitative differences in SUVmax and T/NT, the between mutated and wild-type groups, were obtained using the Mann–Whitney *U* test. The measurement data that did not conform to normal distribution were expressed as medians and the rank-sum test was used for analysis. Moreover, the *Pearson* method was used to calculate the correlation between metabolic parameters. Additionally, the multivariate logistic regression analysis was employed to confirm the predictive value of PET metabolic parameters with regard to the mutational status. Statistical analyses were conducted using the SPSS software, version 22.0 (SPSS Inc., USA) while the ROC curve and predictive value were obtained using the MedCalc software, version 15.2 (MedCalc Software Ltd. Belgium). All analyses were two-sided, and *P* < 0.05 was considered to be statistically significant.

## 3. Results

### 3.1. Patient and Tumor Characteristics

A total of 85 CRC patients were included in this study and the general characteristics of patients are listed in Table 1. All the patients underwent PET/CT scan before primary tumor resection, pathological samples were obtained, and the KRAS/NRAS/BRAF mutational status was evaluated. The majority of the patients were associated with nonmucinous adenocarcinoma (*n* = 83, 97.6%), well/moderate differentiation (*n* = 74, 87.1%), and positive lymph node metastasis (*n* = 67, 78.8%). Among these patients, 56 patients (65.9%) had distant metastatic lesions and 52 (61.2%) were in stage III/IV. Moreover, KRAS/NRAS/BRAF mutations were identified in 46 primary CRC tumors. KRAS mutation was the most prevalent one and was identified in 41 CRC patients (41/85, 48.2%). Additionally, 37 patients had KRAS exon 2 gene mutation type and the mutation rate was 43.5%. The main mutation subtypes were Gly12Asp (*n* = 14, 16.5%), Gly12Cys (*n* = 11, 12.9%), and Gly12Ser (*n* = 7, 8.2%). Furthermore, all the 3 mutations identified in the NRAS gene occurred in exon 3, and the mutation subtypes were Gln61Arg (*n* = 3, 3.5%). Three patients had V600 E mutation in exon 15 of the BRAF gene (*n* = 3, 3.5%). In addition, one of the V600 E mutations in BRAF was accompanied by a mutation in exon 2 of the KRAS gene (Table 2).

### 3.2. Correlation between Metabolic Parameters and the Gene Mutational Status

**Figure 1:**
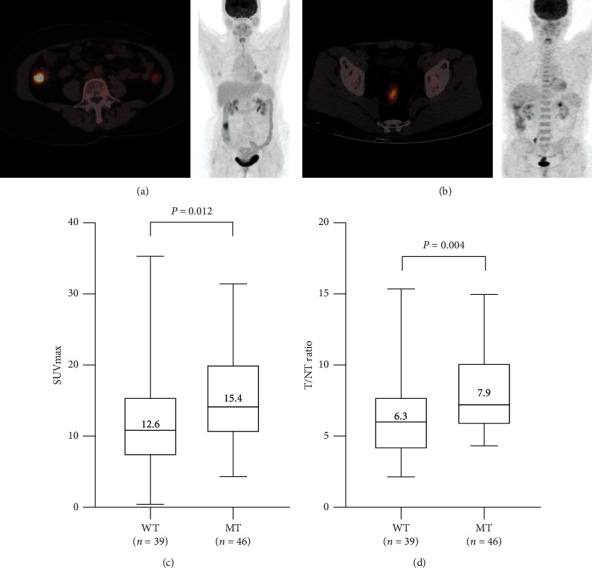
(a) A 60-year-old female with ascending colon cancer with mutated KRAS and wild-type NRAS/BRAF. ^18^F-FDG-PET/CT showed intense accumulation of ^18^F-FDG in the tumor (arrow, SUV: 10.8; T/NT: 6.6). (b). A 58-year-old male had rectal cancer with wild-type KRAS/NRAS/BRAF. ^18^F-FDG-PET/CT scans showed a modest accumulation of ^18^F-FDG in the tumor (arrow; SUV, 4.2; T/NT, 2.6). (c) Analysis of SUVmax according to the status of mutation. The SUVmax of the mutated group was significantly higher than the wild-type group (15.40 ± 6.47 and 12.59 ± 5.79, resp.; *P*=0.0117), in all the primary tumors (*n* = 85). (d) Analysis of T/NT ratios in the two groups. The T/NT ratios of the mutated group were significantly higher than the wild-type group (7.87 ± 2.94 and 6.26 ± 2.83, resp.; *P*=0.0038). WT: wild-type group; MT: mutated group.

The patients were classified into two groups based on the results of KRAS, NRAS, and BRAF mutational status in the primary tumors, including the mutated group (mutation in KRAS/NRAS/BRAF; *n* = 46) and the wild-type group (no mutation; *n* = 39). The clinical characteristics of patients in these two groups are shown in Table 3. The results revealed no significant difference between these 2 groups with regard to gender, age, BMI, tumor location, histologic type, differentiation, T-category, N-category, M-category, primary tumor size, and glucose accumulation in normal liver. However, the two groups were significantly different in glucose accumulation in the primary tumors (Figure 1). The SUVmax was significantly higher in the primary tumors of individuals in the mutated group compared to those in the wild-type group (15.40 ± 6.47 and 12.59 ± 5.79, respectively; *P*=0.012; Figure 1(c)). Also, the T/NT was significantly higher in the primary tumors of patients in the mutated group compared to those in the wild-type group (7.87 ± 2.94 and 6.26 ± 2.83, respectively; *P*=0.004; Figure 1(d)). In a multivariate analysis including factors with a *P* value of 0.35 or less, only SUVmax retained a significant association with the KRAS, NRAS, and BRAF mutations (Table 4; OR, 1.08; 95% confidence interval (CI), 1.01–1.16; *P*=0.028). Similar results were obtained when SUVmax was substituted with T/NT ratio (Table 4; OR, 1.25; 95% CI, 1.05–1.49; *P*=0.012).

### 3.3. The Predictive Value of SUVmax and N/NT on the Gene Mutational Status

**Figure 2:**
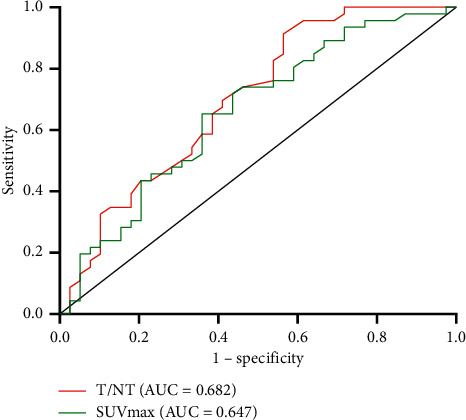
Tumor glucose metabolism parameters for predicting KRAS/NRAS/BRAF mutations. The red and cyan lines represent the ROC curves of the T/NT and SUVmax cohorts, respectively. AUC: area under the curve; SUVmax: maximum standardized uptake value; T/NT: target tissue to nontarget tissue ratio.

The predictive value of SUVmax and N/NT value was evaluated. ROC curve analysis revealed that the highest accuracy (68.2%) was obtained with a particular N/NT cutoff value (Figure 2). At a cutoff value of 12.4, sensitivity and specificity for predicting the presence of KRAS/NRAS/BRAF mutations were 65.2% and 64.1%, respectively (positive predictive value (PPV), 67.4%, 29 of 43; negative predictive value (NPV), 60.5%, 26 of 43; accuracy, 64.7%, 55 of 85). The cutoff value of T/NT 5.1 gave a sensitivity and specificity of 91.3% and 43.6%, respectively (PPV, 64.6%, 42 of 65; NPV, 80.0%, 16 of 20; accuracy, 68.2%, 58 of 85). These results suggested that the ^18^F-FDG PET/CT may be useful in predicting the KRAS/NRAS/BRAF mutational status in primary CRC.

## 4. Discussion

The RAS and RAF family of genes code for proteins that form part of the Ras/Raf/MEK/ERK signaling cascade within cells [21]. The RAS oncogene has three subtypes, namely, KRAS, NRAS, and HRAS. Similarly, the RAF oncogene has three subtypes in mammals, namely, ARAF, BRAF, and CRAF. Mutations in KRAS were shown to occur early in CRC, with an incidence of 30–50% [22]. Additionally, BRAF mutation has an incidence of about 10% [23] and mutation in NRAS at a rate of about 3% [24], while HRAS, ARAF, or CRAF mutations rarely occur [25]. It was reported that mutations in the KRAS gene commonly occurred in exon 2 and Gly12Asp was the codon with the highest rate of mutation [11]. Similar results were obtained in the present study where mutations in exon 2 of the KRAS gene occurred at the highest rate of 43.5% (37/85). Therefore, in this part of the possible KRAS gene mutant patients, clinical medication may have to carefully choose the use of anti-EGFR monoclonal antibody drugs and turn to other drug combinations. We also note that, recently, a drug (AMG510) targeting specific mutations in KRAS has entered clinical trials, but it is only effective in tumors with Gly12Cys mutations in the KRAS gene [26].

In this study, mutations in the BRAF gene were found in 3 patients (3.52%); all of them were V600 E, although this number was lower than that obtained from previous population studies [27]. Our results also showed that mutations in the RAS and BRAF genes did not exist at the same time in a vast majority of the CRC cases, consistent with previous reports [28]. However, there was an exception in a 72-year-old woman in this cohort, who had both KRAS and BRAF mutations, suggesting that the KRAS and BRAF mutations are not always mutually exclusive. Notably, a previous report also showed a rare case of CRC with simultaneous mutations in KRAS, NRAS, and BRAF [29]. Moreover, it was reported that mutations in RAS and BRAF may coexist although this is usually associated with an invasive biology and an adverse clinical course [30]. For instance, the above patient with three concomitant mutations died within a year from the time of his first diagnosis.

The current standard for the detection of gene mutations in CRC is mainly based on the histopathologic analysis. However, this method of examination is often limited by tumor heterogeneity, inconsistency of the gene mutational status, unavailability of tumor tissue, and inadequate sampling. Based on this, studies have tried to investigate the relationship between ^18^F-FDG metabolic parameters and genetic mutations in CRC. For example, Kawada et al. reported that ^18^F-FDG PET may be useful for predicting the KRAS/BRAF mutations with an accuracy of 75%. Additionally, an SUVmax cutoff value of 13 in the analysis of KRAS/BRAF mutations, was shown to give a sensitivity, specificity, positive predictive value, and negative predictive value of 74%, 75%, 71%, and 78%, respectively [16]. Moreover, Lovinfosse et al. [20] reported that rectal cancers with mutations in KRAS or NRAS display significantly higher glucose metabolism compared to the wild types. The SUVmax also showed an area under the curve of 0.65 with a sensitivity and specificity of 69% and 52%, respectively. The present study showed that the two glucose metabolism parameters (SUVmax and T/NT), obtained by ^18^F-FDG imaging, were closely related to the mutational status of KRAS/NRAS/BRAF, and both were independent predictors of gene mutation.

Previous studies explored the potential mechanisms underlying the relationship between glucose accumulation and the KRAS/BRAF mutational status [14–16]. Furthermore, the Warburg effect [31] shows that rapidly proliferating tumor cells need glycolysis to increase energy supply, and the increased expression of glucose transporter 1 (GLUT-1) in tumor cells causes an increase in glucose absorption. It was also reported [32] that hypoxia, KRAS/BRAF gene mutation, and GLUT-1 expression have a synergistic effect and induction of the hypoxia-inducible factor-1alpha (HIF-1*α*) is crucial. However, additional studies [33] also showed that KRAS mutant CRC cells can increase FDG uptake by upregulating the expression of GLUT-1 under normoxic conditions. For example, Yun et al. [34] conducted studies on a variety of CRC cell lines and showed that a low-glucose environment could make the surviving cells acquire KRAS mutations that were not present in their parents, thus upregulating the expression of GLUT-1 and increasing the uptake of glucose.

Moreover, the present study explored the association between genetic mutations in CRC and different clinical characteristics. Such characteristics included gender, age, BMI, tumor location, histologic type, differentiation, T-category, N-category, M-category, primary tumor size, and glucose accumulation in normal liver. The results showed that there was no significant association between these characteristics and mutations in CRC. The study also provided evidence that PET metabolic parameters have an important role in the noninvasive prediction of the KRAS/NRAS/BRAF mutational status in CRC. Notably, SUVmax is the most commonly used metabolic parameter in PET and varies with different factors, including the type of PET scanners, metabolic differences among patients, and plasma glucose levels after fasting duration. However, T/NT is a weighted value, which reduces the interference of the above factors by comparing with the patient's own nontumor tissue. Therefore, T/NT may be superior to SUVmax in predicting the gene mutational status.

While the study uncovered some insightful findings, it had a number of limitations. First, this was a retrospective study conducted by a single team and might have had a bias. Therefore, the findings should be confirmed externally using different scanners, resolution settings, and reconstruction algorithms. Second, PET scan reflects the general state of the tumor, while the heterogeneity of CRC intratumoral mutations may have affected the correlation analysis because the anatomical specimens for the detection of mutations detection may not have accurately reflected the macroscopic state of the entire tumor. Third, the study included all four stages of CRC patients, similar to other studies [11, 16]. However, it might have been better to include only stage IV patients because of less intratumoral heterogeneity in genetic mutations [35]. Another possible shortcoming of this study is the relatively small number of samples used; therefore, larger sample size is required for future systematic studies. Moreover, the imaging features obtained by PET/CT are still not sufficient to replace the standard methods of detecting mutations in CRC because of the relatively low predictive specificity and accuracy obtained.

## 5. Conclusion

In this study, the accumulation of ^18^F-FDG was higher in CRC tumors harboring KRAS/NRAS/BRAF mutations. Additionally, T/NT and SUVmax could be surrogate imaging indicators useful for the analysis of tumor genotypes in CRC. However, the two indices warrant further studies because of the relatively low predictive accuracy.

## Figures and Tables

**Table 1:** Patient and tumor characteristics.

Characteristics	Cases (%)
Patients, *n*	85

Gender	
Male	51 (60%)
Female	34 (40%)

Age, years	
Mean ± SD	59.27 ± 11.56
Range	26–79

BMI	
Mean ± SD	21.97 ± 3.21
Range	15.43–33.87

Tumor location	
Left hemicolon	33 (38.9%)
Right hemicolon	22 (25.9%)
Rectum	30 (35.2%)

Histologic type	
Nonmucinous adenocarcinoma	83 (97.6%)
Mucinous adenocarcinoma	2 (2.4%)

Differentiation	
Well/moderate	74 (87.1%)
Poor	11 (12.9%)

UICC-TNM stage	
I/II	33 (38.8%)
III/IV	52 (61.2%)

T-category	
Tis, T1, T2	4 (4.7%)
T3, T4	81 (95.3%)

N-category	
Positive	67 (78.8%)
Negative	18 (21.2%)

M-category	
Positive	56 (65.9%)
Negative	29 (34.1%)

Tumor size, mm	
Mean ± SD	49.2 ± 28.17
Range	10–180

Mutational status	
KRAS/NRAS/BRAF mutated	46 (54.1%)
Wild-type	39 (45.9%)

UICC, Union for International Cancer Control.

**Table 2:** Distribution of genetic mutations in the primary CRC tumors (*n* = 85).

Gene mutational status	Cases (%)
Mutated group (mutation in KRAS, NRAS, or BRAF)	46 (54.12%)
Mutated KRAS only	40 (47.06%)
Mutated NRAS only	3 (3.53%)
Mutated BRAF only	2 (2.35%)
Mutated KRAS and BRAF simultaneous	1 (1.18%)
Wild-type group (no mutation)	39 (45.88%)

**Table 3:** Univariate analysis of factors associated with the KRAS/NRAS/BRAF status.

Factors	Mutated group	Wild-type group	*P*
Gender			0.657
Male	25	26	
Female	21	13	

Age, years			0.249
>59	23	27	
≤59	23	12	

BMI			0.342
Mean ± SD	21.96 ± 3.57	21.98 ± 2.78	

Tumor location			0.288
Left hemicolon	17	16	
Right hemicolon	14	8	
Rectum	15	15	

Histologic type			0.711
Nonmucinous adenocarcinoma	45	38	
Mucinous adenocarcinoma	1	1	

Differentiation			0.497
Well/moderate	39	35	
Poor	7	4	

UICC-TNM stage			0.406
I/II	16	17	
III/IV	30	22	

T-category			0.421
Tis, T1, T2	0	4	
T3, T4	46	35	

N-category			0.354
Positive	38	29	
Negative	8	10	

M-category			0.534
Positive	35	21	
Negative	11	18	

Tumor size, mm			0.406
Mean ± SD	49.87 ± 27.11	48.41 ± 29.70	

SUVmax in tumor			0.012
Mean ± SD	15.40 ± 6.47	12.59 ± 5.79	

SUV in normal liver			0.597
Mean ± SD	1.97 ± 0.43	1.87 ± 0.36	

T/NT			0.004
Mean ± SD	7.87 ± 2.94	6.26 ± 2.83	

UICC: Union for International Cancer Control; SUVmax: maximum standardized uptake value; BMI: body mass index; T/NT: target lesion to nontarget lesion ratio.

**Table 4:** Multivariate analysis of the KRAS/NRAS/BRAF status in patients with CRC (*n* = 85).

Factors	OR (95% CI)	*P*
Age	0.98 (0.95–1.02)	0.420
BMI	1.03 (0.89–1.20)	0.664
Tumor location	2.06 (0.63–6.67)	0.231
SUVmax	1.08 (1.01–1.16)	0.028
Age	0.98 (0.94–1.02)	0.402
BMI	1.04 (0.89–1.22)	0.578
Tumor location	2.19 (0.66–7.33)	0.203
T/NT	1.25 (1.05–1.49)	0.012

OR: odds ratio; CI: confidence interval; BMI: body mass index; SUVmax: maximum standardized uptake value; T/NT: target lesion to nontarget lesion ratio.

## Data Availability

The data used to support the findings of this study are available from the corresponding author upon reasonable request.
